# Exploiting Big Data for Experiment Reporting: The Hi-Drive Collaborative Research Project Case

**DOI:** 10.3390/s23187866

**Published:** 2023-09-13

**Authors:** Alessio Capello, Matteo Fresta, Francesco Bellotti, Hamed Haghighi, Johannes Hiller, Sajjad Mozaffari, Riccardo Berta

**Affiliations:** 1 Department of Electrical, Electronic and Telecommunication Engineering (DITEN), University of Genoa, Via Opera Pia 11A, 16145 Genoa, Italy; 2 WMG, University of Warwick, Coventry CV4 7AL, UK; 3 Institute for Automotive Engineering (IKA), RWTH Aachen University, Steinbachstr. 7, 52074 Aachen, Germany

**Keywords:** big data architecture, project monitoring and reporting, non-relational DB, RESTful APIs, field operational tests, automated driving

## Abstract

As timely information about a project’s state is key for management, we developed a data toolchain to support the monitoring of a project’s progress. By extending the Measurify framework, which is dedicated to efficiently building measurement-rich applications on MongoDB, we were able to make the process of setting up the reporting tool just a matter of editing a couple of .json configuration files that specify the names and data format of the project’s progress/performance indicators. Since the quantity of data to be provided at each reporting period is potentially overwhelming, some level of automation in the extraction of the indicator values is essential. To this end, it is important to make sure that most, if not all, of the quantities to be reported can be automatically extracted from the experiment data files actually used in the project. The originating use case for the toolchain is a collaborative research project on driving automation. As data representing the project’s state, 330+ numerical indicators were identified. According to the project’s pre-test experience, the tool is effective in supporting the preparation of periodic progress reports that extensively exploit the actual project data (i.e., obtained from the sensors—real or virtual—deployed for the project). While the presented use case concerns the automotive industry, we have taken care that the design choices (particularly, the definition of the resources exposed by the Application Programming Interfaces, APIs) abstract the requirements, with an aim to guarantee effectiveness in virtually any application context.

## 1. Introduction

Managing projects necessitates controlling their timing and cost in an effort to meet planned targets. In order to take the most appropriate actions, the management team needs timely data representative of the project’s state [1]. Thompson et al. [2] have suggested that flawed status reporting is a serious concern in information system projects. Iacovou et al. [3] showed a positive effect of reporting quality on project performance and indicated that selective reporting behavior (e.g., optimistic biasing) has a degrading effect on reporting quality. We expect that advances in big data technologies may lead to improvements in this area of project progress monitoring and reporting. However, there the literature is lacking with regard to tools specifically dedicated to project monitoring exploiting big data end to end, from the experimental/project data files up to the key performance indicators (KPIs) and their visualization and management. Data files contain the raw data logged by the sensors deployed by a project/experiment. Thus, we propose a further exploitation of such sensors not only for the aims of the project, but also for quantitative reporting, aiming at improving its coherence, accuracy and efficiency.

This observation prompted us to investigate the exploitation of state-of-the-art big data management methods and tools to improve project status monitoring and reporting. Our research is also interested in characterizing the main features of a periodic reporting system, independent of the application domain, as well as what types of metadata may be used, and what user workflow should be supported and the relevant modalities of human–computer interaction (HCI).

The context and motivation of this research come from the automated driving (AD) industrial sector, which represents a highly significant investigation domain given the huge amount of research that is being carried out in the field (e.g., [4,5,6,7]). In the L3Pilot project [8], dedicated to piloting SAE level 3 automated driving functions (ADFs), we studied how to organize a robust workflow for quantitatively addressing research questions (RQs) in a collaborative project sharing sensitive data among various partners, while ensuring methodological soundness and data validity, and protecting partners’ intellectual property (IP) [9,10]. This process was driven by a well-established reference methodology for large scale pilots and field operational automotive tests, namely Field opErational teSt supporT Action (FESTA) [11]. Data are at the center of the methodology, as they are the basis for the analysis and assessment steps, which provide the final outcome of the project. Consequently, a data toolchain was designed to support an efficient and effective test phase, where data are collected, and assessment phase, where data are analyzed. A key novelty in order to obtain the quantitative information needed to answer the L3Pilot RQs was the development of the Consolidated Database (CDB), based on the open source Measurify technology [12], which allows data from all the pilot sites to be shared anonymously and securely amongst project partners to facilitate data analysis aimed at answering the project’s RQs.

Continuing the L3Pilot perspective, the Hi-Drive project [13]—EUR 30 M funding, with a consortium of 40 partners among original equipment manufacturers (OEMs), suppliers, user associations and research organizations—strives to extend the operational design domain (ODD) of ADFs and reduce the frequency of their takeover requests (TOR), by means of a set of new technology enablers aimed at enhancing performance of state of the art ADFs. Passenger cars and trucks will demonstrate this in a large set of traffic environments, with a specific attention to demanding, error-causing conditions.

Similarly to L3Pilot, the Hi-Drive’s plan involves a set of experiments that are being conducted in several countries. In Hi-Drive, an experiment is defined as an entity consisting of a series of test vehicle runs/trips aimed at investigating a specific use case (e.g., lane merging, non-signalized intersection crossing) and is conducted under comparable circumstances. Experiments are expected to last between 6 and 18 months. Every experiment tests vehicles from one or more vehicle owners (i.e., OEMs or suppliers), whose ADFs have been enhanced by one or more enablers. Hi-Drive enablers are technological tools (hardware, software or methodology) that have the potential to enable new ADF(s) and/or upgrade existing ADF(s). Hi-Drive enablers concern technologies such as connectivity, positioning, cyber-security and machine learning. Experiments compare the impact of enabler-enhanced prototypes with a baseline that is given by an ADF without the corresponding enabler(s). The project considers different types of impacts: safety, efficiency and environmental, transport system, mobility, socio-economic and user acceptance.

Given the strong similarities of the experimental processes, the Hi-Drive reference architecture builds on the L3Pilot data architecture [9,10]. However, a critical assessment of the L3Pilot experience stressed the need for improving the periodical reporting on the state of the project in the different pilot sites, which suffered difficulties in extracting and managing KPIs (e.g., how many kilometers were travelled in the period, in what road type; how many persons took part in the tests, with what roles, etc.). The experience thus suggested the idea of exploiting the huge quantity of the project’s experimental data with a view to having a homogeneous quantitative reporting from all the pilot sites, according to a common schema that was defined in the initial phase of the project. This led to the idea and design of the Experimental Metadata Database (EMDB), an end-to-end big data management application dedicated to supporting reporting and enabling the project management to obtain an overview of all conducted experiments. The project managers (e.g., project coordinator and work-package/task leaders) should be able to adequately track the progress of the activities and easily analyze the experiments’ state, through a sort of “dashboard of the project”.

In the design, we made sure to generalize requirements and devise abstract solutions, so to build a generic tool that could be easily configured also for other projects and application domains.

This paper presents the challenges we have faced in developing the tool, the implemented design solutions and the feedback from use in the project. The remainder of the article is organized as follows. The next section presents the related work. Section 3 illustrates the requirements elicited for the system, while Section 4 details the technical implementation. Section 5 presents and discusses the achieved results, while Section 6 draws the conclusions on the work conducted.

## 2. Related Work

The importance of data has grown exponentially in recent years, with the development of technologies for dealing with big data sensing, processing, management, and mining (e.g., [14,15,16,17]). Many data analytics tools have been developed to assist in specifying, integrating, and deploying data analytics applications. Khalajzadeh et al. [18] presented a survey and analysis of several current research and practice approaches to support data analytics for end-users. Ataei and Litchfield [19] conducted a systematic literature review on big data reference architectures. They highlighted major challenges for research such as micro-services architectures, event-driven paradigms, security and privacy issues, and metadata management. Github provides good examples of templates, typically in LaTeX, for scientific experiment reporting (e.g., [20,21]).

However, the literature lacks information about tools for the use of big data to support experiment/project state reporting. The concept of a Project Consortia Knowledge Base (PC-KB) is presented by Winkler et al. [22] in an integration framework based on semantic knowledge that facilitates project-level communication as well as access to project data across tool and partner boundaries. Theories and technologies related to knowledge discovery have been applied to all kind of databases because of their abilities in converting raw data into useful knowledge for operation management, decision making and in-depth analysis and reporting. For instance, Hsu and Ho [23] developed a knowledge discovery model using a data warehouse technique to facilitate data gathering and in-depth analysis and news reporting.

On the other hand, there is significant bibliography stressing the importance of objective reporting.

Recent research has suggested that flawed status reporting is a serious concern in Information System (IS) projects. The linkage between reporting quality and project performance was empirically studied by Thompson et al. [2] in two studies, with 210 IS project members and 485 IS project managers, respectively. Both studies showed that the perceived quality of project reporting was improvable and significantly associated with project task outcomes. The second study suggested that reporting quality was also related to organizational outcomes.

In a survey of 561 project managers, Iacovou et al. [3] investigated selective reporting behaviors that are pursued by project managers when communicating the state of their information system initiatives to their executives. The findings of the study reveal a positive effect of reporting quality on project performance and indicate that a specific type of selective reporting behavior (optimistic biasing) has a degrading effect on reporting quality.

El-Omari and Moselhi [1] presented a control model that integrates different automated data acquisition technologies to collect data from construction sites for progress measurement purposes. The paper discusses suitability of various automated data acquisition technologies (e.g., bar coding, Radio Frequency Identification (RFID) 3D laser scanning, photogrammetry) for tracking and controlling construction activities. The authors propose a cost/schedule control model which integrates automated data acquisition technologies with a planning software module, a relational database, and AutoCAD to generate progress reports that can assist project management teams in decision making.

In the automotive industry, a number of field operational tests (FOTs) have been executed in recent years to test new advanced driver-assistance systems (ADAS) in authentic traffic conditions, involving thousands of drivers (e.g., euroFOT [16,17]). The FESTA methodology covers the whole process of planning, preparing, executing, analyzing and reporting an FOT. The steps that need to be carried out during an FOT are graphically presented in the form of a “V”-shape diagram, where there is “horizontal” correspondence between the levels on the left-hand side (concerning the “preparation” of the FOT) and right-hand side (concerning the “analysis” or “evaluation”). The left side of the “V” descends from definition of functions and use cases, down to research questions and hypotheses, performance indicators and measures, data collection tools and pilot site set-up. The bottom of the “V” is given by data acquisition during tests. The right side of the “V” rises mirroring the left side: data processing, data analysis to answer research questions on technical performance, user acceptance and behavior, impact on traffic and mobility, up to societal impacts. The L3Pilot project adopted the FESTA methodology and built its data toolchain to support it, particularly answering four sets of research questions, concerning impact on technical performance, user acceptance, traffic, and society. Through the EMDB, Hi-Drive extends this solution also to the case of quantitatively supporting periodical project/experiment reporting.

## 3. Specifications and Requirements

This section reports, in the next two sub-sections, respectively, the reporting data specifications and the design requirements that were elicited in the early phases of the project. The former were established by the Hi-Drive’s Methodology sub-project, also based on the experience in previous projects. The latter were collected and then harmonized in a page on the project’s online collaboration tool, where partners could detail one or more specific needs, in natural language, together with a short user story illustrating the related application and advantages. In a user-centric design perspective, this list of requirements was further complemented with the feedback from early lab tests and demos with partners, which informed useful design iterations.

### 3.1. Data Specifications

For specifying the data needs, a schema was defined in the initial phase of the project to make all the experiments report their state in a quantitative and consistent way, considering the variety of settings across the different experiments.

The schema is encoded in an Excel sheet (an excerpt of the file is provided in Table 1), which gives detailed structure and specification (until the level of the type of the single fields) of the signal list (i.e., the data expected to be stored in the EMDB). The sheet has one row for each unit of data (namely, indicator) quantifying the project/experiment progress in a particular aspect. In a hierarchical approach, which was deemed appropriate to deal with their huge number (330+) and variety, indicators are grouped into 35 topics. Examples of topics include the following:

- Duration of the experiments, which contains, as related indicators, the number of tests lasting up to 15 min, between 15 and 30 min, 30 and 60 min, etc.;
- ADF operation times, which includes the number of activations with operation time less than 1 min, between 1 and 5 min, between 5 and 10 min, etc., and the number of encountered traffic sections (e.g., pedestrian crossings, roundabouts, traffic lights, tunnels);
- Travelled distance and driving times, which are in turn segmented in terms of the time of day (e.g., morning, afternoon, evening, night), type of road (motorway, urban, rural, etc.), road conditions, number of lanes, speed limits, state of the ADF and enabler (activated or not).

**Table 1:** Excerpt of data requirements Excel file.

Covered Information		Description
Group	Specific Value	
Time of tests in the experiment	Between 0 and 6	Number of test runs performed between hour x and hour y, local time.
	Between 6 and 12	
	Between 12 and 18	
	Between 18 and 24	
Duration of tests in the experiment	Less than 15 min	Number of test runs per-formed during the indicated length of time.
	Less than 30 min	
	Less than 1 h	
	Less than 1 h and 30 min	
	Less than 2 h	
	Longer than 2 h	

A key requirement due to the huge number of indicators to be periodically reported according to the above mentioned data specifications. Since providing all this data manually at each reporting period would be overwhelming, it was required to develop scripts that could extract most of the indicator values from the raw files used in the experiments.

### 3.2. User Requirements

The main requirement that emerged during the initial analysis and design steps was system configurability, especially in terms of experiment descriptors and indicators. Support for configurability was intended to facilitate dealing with possible extensions during the actual development, and also allow the possibility of using the tool in other domains as well. To this end, abstraction was a key design factor. For instance, we generalized the concept of Experiment as a generic project activity, so that it could be applied to other contexts as well, without sticking to domain-specific characteristics.

Other requirements emphasized the importance of user-friendliness and, thus, demanded the availability of a graphical user interface supporting all the data management operations according to the different typologies of users involved. Overall, project partners required support for understanding the system (e.g., through hands-on workshops) and during the actual operations.

Requirements mandated that all the graphical user interfaces (GUIs) be implemented as cloud-based applications accessible from common browsers, to prevent the burden of local executable installations (avoiding platform-dependent compatibility issues and security locks in a company’s PCs). This ensures that all the partners can seamlessly employ the latest version of the application. On the downside, this approach limits the interface flexibility and modalities of local file accessibility, but this did not compromise overall effectiveness.

Data integrity is obviously a key requirement. As we will see later, this is targeted through the utilization of schemas that mandate the structure of each datum and the employment of a suitable design pattern (particularly, the “Protocol-Experiment” pattern).

Data confidentiality concerns the fact that experiment managers should be able to upload and access data about their own experiment, but not those of the others, while project managers should be able to read data from all the experiments.

The functioning of the EMDB server itself does not imply strict performance requirements, given the relatively limited amount of data (in the order of MBs) to be uploaded by each experiment manager (the expected number of experiments is 20/30) and downloaded by project managers. However, generalizing the specific use case, the solution is big data ready, being based on MongoDB, a popular (particularly, involving a large, active community of users and rich documentation), high-performance, state-of-the-art non-relational DB system [24,25]. On the other hand, huge quantities of data are processed by the toolchain at each experiment’s site, for the automatic extraction of the progress/performance indicators from the project’s raw files.

## 4. System Design and Implementation

With regard to the platform for the data management in Hi-Drive, the choice fell on Measurify, a cloud-based, open source, abstract, measurement-oriented data framework for managing smart things [12,26,27]. Besides its measurement-orientation, the reasons for the choice concern the fact that Measurify is platform independent and non-cloud vendor locked, which implies a straightforward portability across partners’ and cloud providers’ infrastructures. Through high levels of abstraction, the framework supports an easy and efficient development of data-rich applications, particularly by supporting the instantiation of application databases (ADB, i.e., a Measurify instance configured for a specific application) by simply editing configuration files, without writing software. Measurify was developed and employed also in L3Pilot, where it was used to implement, as ADBs, the Performance Indicator DB and the User Questionnaire DB [10]. In Hi-Drive, we decided to also implement the EMDB as a Measurify ADB. Besides the normal configuration, this also required the development of some extensions (particularly, the Experiment and Protocol resources), which are described in the following sub-section. Before that, to set the proper background, we outline the main characteristics of Measurify.

Measurify is implemented in NodeJS and features a RESTful API. It relies on MongoDB as the underlying database management system. Measurify ADB configuration is achieved by populating the fundamental resource collections with the application-specific values. Table 2 synthetizes the main resources available in the framework. A Feature resource fully and formally describes the data types of the Measurements to be uploaded and managed by the ADB. This resource is used for the data integrity check at each Measurement upload. The MongoDB non-relational DB in fact supports performance and development efficiency but needs the utilization of schemas to guarantee content integrity. Thus, the Measurify’s Feature resource has been designed to provide the schema to which all the Measurements referring to that Feature in the DB must comply. Every measurement must refer to a Feature. This is called the Measurement–Feature pattern.

Data integrity is obviously a key requirement for the Hi-Drive DBs. Compliance is guaranteed through the utilization of schemas, that mandate the structure of each piece of data, and the employment of the Measurement–Feature pattern. Since measurements can be of different high-level data types, this pattern requires that each measurement references its specific type (i.e., Feature), and thus, its structure is checked against its declared reference before being stored in the DB.

### 4.1. Extensions to Measurify

The main requirements for the EMDB design process were essentially encoded in an Excel file, which provides a detailed structure and specification of the data expected to be stored (Table 1). The raw data in this category are mostly single values (either numeric or string), but storage of histograms (e.g., of driven velocities) is also required, thus demanding the use of arrays.

Analyzing the specifications from an end-user’s perspective, we observed that every experiment contains three types of information entries:

- A few basic **descriptor** fields, such as name of the experiment, location, managing organization, start and end date, etc.;
- Several **metadata** items for more in-depth description, such as type of targeted enablers (e.g., vehicle to infrastructure V2I, localization, positioning, machine learning, etc.), technical focus (e.g., technical enablers, users, etc.), etc.;
- State-tracking, progress/performance **indicators** (e.g., number of baseline tests/runs performed for this experiment, number of tests performed per time of day, per experiment length, travelled distance with ADF and enabler activated, etc.).

In the above, on the one hand, the descriptor fields and metadata describe the nature (static properties) of the experiment. Descriptors are basic and common to all types of experiment, while metadata are specific to the experiments of a given project. On the other hand, state-tracking indicators are to be periodically updated by the experiment managers during the execution of an experiment. Thus, they represent the dynamic properties of an experiment. The indicators’ values constitute the experiment’s history, which is a chronology of records reporting the state of the experiment in given steps in time, which should be useful for the project and site managers, for instance to prepare charts and/or tables. 

Each history record to be uploaded to the DB includes an ordinal number, namely Step, that indicates the step in the history to which this record refers. The step can seamlessly (and arbitrarily) refer to months, quarters, years, etc. The first (logical) progress is indicated as step 1, the second one as step 2, etc. This allows both the insertion of new history steps and (if needed) updates of old history steps. The history indexing strategy is completely up to the user, and it is argued, but not mandated, that experiment managers in a single project (e.g., Hi-Drive) should coordinate among each other to follow a consistent approach. 

In order to take account of possible future changes and extensions, we have defined the concept of Protocol. A protocol specifies the experiment’s type. To each experiment, in fact, a protocol must be associated, which specifies all the information (i.e., descriptors, metadata, indicators, and their relevant types) that will be recorded for that experiment. Both Protocol and Experiment have been implemented as Measurify resources and are now available for any kind of project. 

For Hi-Drive, at least based on the current specifications, all the experiments refer to only one Protocol (namely, the “Hi-Drive” protocol resource). However, the use of different, customized protocols is an abstraction that may also be used within a single project involving different types of experiments, to help users to focus only on the type of data relevant to their particular experiments. 

The Protocol resource specifies the names and data types of the Metadata and the History records usable by the experiments that refer to that protocol. Thus, the Experiment–Protocol pattern is analogous to the Measurement–Feature pattern, with respect to experiment-related information. 

History records are hierarchically structured into Topics and Fields, which are ‘the children’ of Topics, and represent the above mentioned progress/performance indicators. This hierarchical structure corresponds to the taxonomy specified in the above-mentioned specifications file. For instance, the Road Condition topic is subdivided into several fields, such as Road_dry, Road_wet, Road_icy, Road_snowy, etc. An experiment state at a given time step is given by the value of such fields. 

**Figure 1:**
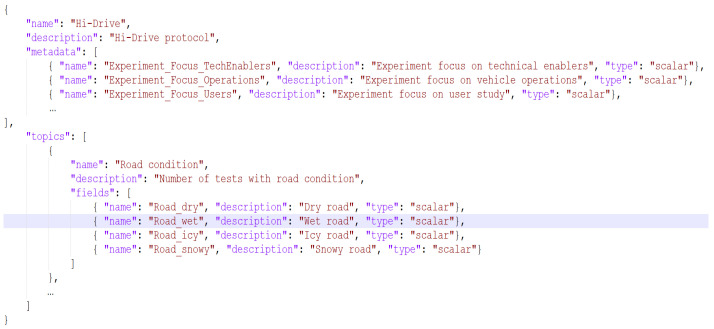
Excerpt from the Hi-Drive Protocol specified in .json.

The whole Hi-Drive Protocol resource in Measurify is specified in a .json file (“*Protocol Hi-Drive.json*”). This file is not exposed to the EMDB users, but only to administrators, as it specifies the data types to be provided by the experiment. What an end-user sees, as we will show in Section 4.3 “Graphical User Interface”, is a web-form or a .csv file displayed/formatted according to the protocol. A very limited excerpt of the “Hi-Drive” protocol resource is provided in Figure 1 below.

**Figure 2:**
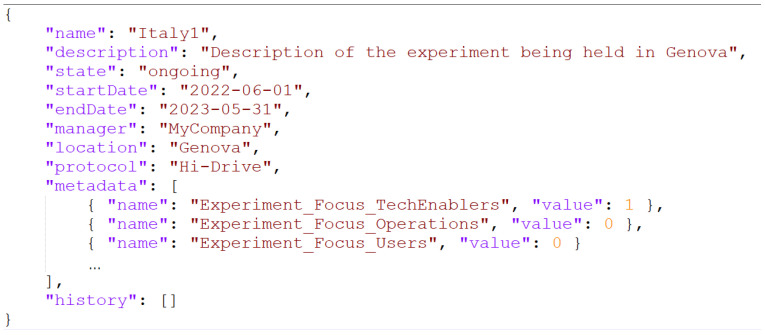
Excerpt of Experiment Italy1.json.

An example, minimal and dumb (for confidentiality reasons), of Experiment configuration file using the Hi-Drive protocol is provided in “Experiment Italy1.json” (Figure 2). As anticipated, the file specifies the values for this experiment of the basic descriptor fields (included the reference protocol) and of the contextual data (i.e., the metadata). A similar file is to be prepared by each experiment manager before the start of the experiment. As can be seen, the history list is initially empty.

The file “Italy1#step2.csv” (an excerpt of which is reported in tabular form in Table 3), instead, is an example of a possible History step record that could be uploaded by an experiment manager. The example reports the number of test runs conducted in the second step of the experiment, segmented by road condition. Through a convention on filenames, the toolchain is able to detect the proper experiment’s history to update.

**Figure 3:**
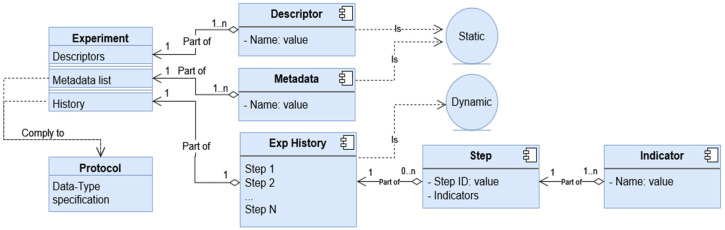
Organization of the data structure for an experiment.

As a summary (Figure 3), the information to be provided for each experiment has been mapped following these criteria:

- Very basic information about the nature of the experiment is mapped to the Experiment Descriptor fields. This data structure is common to all the experiments/projects;
- More detailed information about the nature of the experiment is mapped to the Experiment Metadata. This data structure is experiment specific;
- The indicators periodically reporting the progress of the experiment are mapped to the Experiment History record. This data structure is experiment specific.

Finally, it is important to highlight the generality of the proposed design, since Descriptors, Metadata and History are concepts applicable to virtually any type of experiments/projects, and the Protocol resource abstraction allows this flexibility to be implemented with intrinsic data integrity checking.

### 4.2. The EMDB Workflow

According to the Measurify model [12], the workflow consists of two main phases:

1. Configuration, which sets up the specific ADB;
2. Operation, which fills it during the progress of the application.

**Figure 4:**
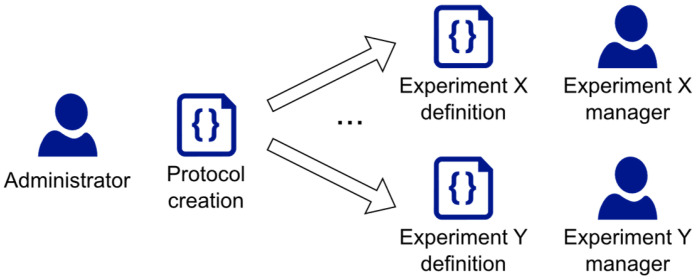
Steps for the creation and initialization of the EMDB.

In the first configuration step, the EMDB administrator creates the Protocol resources in the EMDB. In the Hi-Drive case, there is only one resource, which is instantiated by uploading the already presented “Protocol Hi-Drive.json” file. Then, each Experiment manager defines his own Experiment in a .json file and uploads it to the EMDB (“Experiment Italy1.json” is a configuration file example, shown in Figure 2). The EMDB configuration steps are depicted in Figure 4.

**Figure 5:**
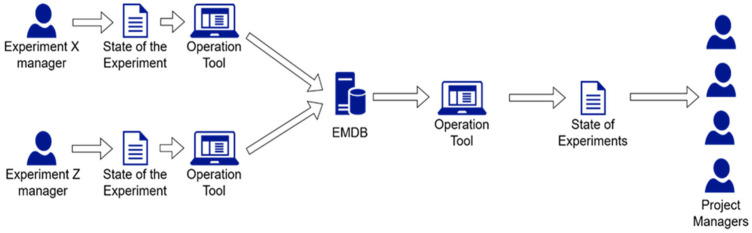
Typical steps for the operation phase of the EMDB.

Once configured, the EMDB is ready for receiving data from the various test sites. Each experiment manager can report the state of the experiment at a given history step by uploading a file (e.g., “Italy1#step2.csv” shown in Table 3) through the Operation Tool, a graphical user interface which will be described in the next subsection. Then, project managers can retrieve the state of all the Experiments present in the ADB. Experiment managers are allowed to update and retrieve data about their Experiment(s), while Project managers are allowed to retrieve data of all Experiments. Figure 5 provides an overview of the operation phase of the EMDB.

### 4.3. Graphical User Interface

Given the relevance of the partners’ requirements, special attention was paid to the graphical user interface (GUI). The GUI consists of three tools that are available as simple web applications on browsers: the Admin Dashboard, the Operation Tool and the Data Visualizer. 

**Figure 6:**
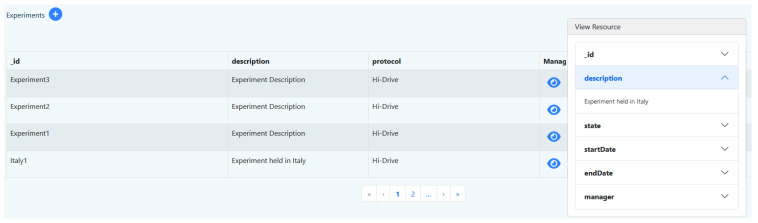
Outlook of the Admin Dashboard page for managing experiments.

The Admin Dashboard has been designed to support DB administrators to create and initialize (i.e., configure) their ADBs. The Admin Dashboard supports creation and configuration of all the Measurify resources, including Protocols and Experiments (e.g., Figure 6). Thus, the .json files needed for configuring of an EMDB installation (including the single experiments) can be set up by filling in fields in a visual form, if preferred by the user.

The Operation Tool is the tool through which the various DB users can perform the typical data upload and download operations to/from an ADB. The Operation Tool is designed to support the operations of the workflow presented in Figure 5.

The Data Visualizer offers a user-friendly way for experiment and project managers to quickly obtain a graphical overview of the EMDB data. The GUI supports bar- and pie-type interactive charts. The bar chart displays the absolute values of each item within the selected data group (a data group is one of the 35 topics defined in Section 3.1), while the pie chart shows their relative values as percentages. If a data group consists of a single item, the charts will be generated for different steps of the experiment instead of different items within the group.

These tools have been developed using state-of-the-art JavaScript libraries for building advanced user interfaces, such as React [28], Vue.js [29], and Chart.js [30].

### 4.4. Automatic Extraction of the Progress/Performance Indicators

As per the requirement of reducing the manual effort needed to fill in the periodic reports to be inserted in the EMDB, we designed Python scripts to extract the progress/performance indicators. Extraction is achieved by processing the .hdf5 files that log the timeseries of the signals obtained by the vehicular sensors (e.g., speed, pedal activity, ADF activity, etc.), that are enriched in post-processing with derived/context measures (e.g., detected driving scenarios, relative position of other traffic actors, type of road, weather conditions, etc.) [10]. Since one .hdf5 file is created for each test session (i.e., test-vehicle’s trip), the script that takes in input a directory containing all the .hdf5 files recorded in the reporting period and outputs the .csv file to be uploaded to the EMDB, with most of its fields automatically filled. The script associates a counter to each fillable indicator. Some of these counters (e.g., number of tests on wet roads) are simply incremented by one for each .hdf5 file meeting the relevant criterion. Other counters (e.g., number of take-over requests) are incremented per each .hdf5 file by the value obtained by processing the relevant timeseries in that file. A few other indicators, which are not related to values logged during a trip (e.g., number of trip participants, age, etc.), cannot be computed automatically and are left to be manually filled by the experiment manager. 

**Figure 7:**
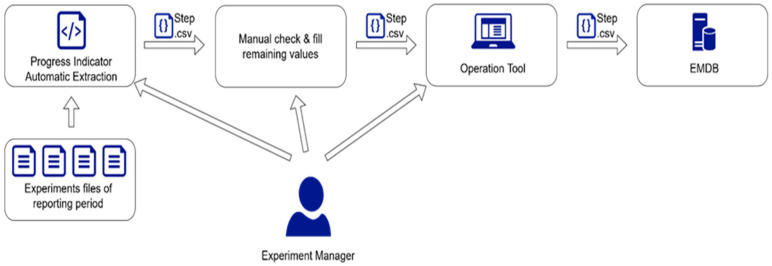
Periodic report preparation process.

Figure 7 provides an overview of the periodic reporting process. The experiment manager collects all the relevant files in a root directory, executes the automation script, checks the resulting .csv file, manually fills the missing values, and finally uploads the file to the EMDB via the Operation Tool on a browser.

### 4.5. Lab Testing

The system architecture has been implemented with a systematic exploitation of automatic unit testing to aim at achieving robustness and guarantee that each version release could meet the requirements. This is particularly important in languages which are not strongly typed, such as JavaScript, so properties or methods of variables may depend on inputs. Thus, the scope of static verification is limited, and actual checks should be performed at runtime. Unit tests have been prepared for all the functionalities provided by the APIs, and new releases are made only after all unit tests have been successfully passed. Testing of the server functionalities can be performed through the GUI or directly through API testing routines directly built into Measurify, which is the way employed for the automatic unit testing.

The client’s GUI has been verified in lab tests and discussed in demos with the relevant partners. Automatic unit tests have been set up also for the GUI, using the Cypress.io tool [31], which proved itself very useful as an independent test runner that does not need to closely integrate with source code. Through an interactive web page accessible from the browser, the developer can visually execute sequences of actions on the GUI under development. Each one of such sequences is a unit test that is recorded by Cypress.io to be later employed in automatic testing.

## 5. Results and Discussion

### 5.1. Deployment

Lab tests were useful to release a stable system implementing the functionalities according to the requirements specified in Section 3 “Specifications and Requirements”. However, as is common in research projects, design iterations are needed (and reasonably expected), based on the feedback of the actual end-users using early versions of the system under development. To this end, demos and then hands-on workshops were organized since the early phases of the project, in order for partners to familiarize themselves with the workflow. The availability of a good tutorial, to be followed at the workshops, is essential. Then, a pre-pilot phase was organized to deploy and test the systems to verify their overall functioning before the actual execution of the tests that will produce the final project results. Feedback is requested by all the partners during the pre-tests in order to allow the system to achieve the desired level of service. Pre-tests concern 20 operational sites.

Two solutions are requested and implemented for the deployment of the Hi-Drive DBs: (i) in the cloud; (ii) on premises. In both cases, users are able to manage data through the previously presented GUI. The former solution is particularly suited for project-level DBs but could also be used for EMDB instances available to single partners only. To this end, the Hi-Drive cloud space on Amazon Web Server (AWS) was set up, hosting an EC2 t2 medium machine dedicated to the pre-pilot EMDB with Ubuntu operating system, which can be scaled according to further requirements.

The on-premises EMDB solution has been thought of typically for private installations, in which partners can have local tests with their own data, before sharing them at consortium level in the cloud. Moreover, they may try different local DB configurations (e.g., with new Features implementing different data types), for instance, for exploring possible solutions that could be later extended to the whole consortium. This requires that partners download the source code and proceed with the installation of the server and then the configuration of the EMDB, as described in the previous section.

The actual experience in the pre-pilots is showing a very limited use of on-premises deployment, while the cloud solution, in which installation is managed by the project-level DB-administration team, is more convenient, also for single-partner EMDBs.

Pre-pilot tests have been very useful in allowing partners to gain practical experience with all the modules developed during the project and thus provide concrete suggestions on how to improve their usability. For the EMDB, this implied several tweaks in the GUI design (e.g., position and text of the widgets, consistency among the web pages, feedback messages to the user, content of the tutorial/user manual web pages). 

A major outcome from the pre-pilot tests concerns verification of efficiency both in deployment and usage. This concerns both the experiment set-up time and the history update time. 

The developed system allows experts to focus on experiment description by defining the two lists of metadata and of progress indicators as .csv files. The definition of the corresponding data types (Protocol resource, one for the whole Hi-Drive project) is straightforward. Once the files are ready, the EMDB is instantiated in few seconds.

Table 4 gives a summative quantitative characterization of an EMDB cloud installation targeted for Hi-Drive. Table 5 shows the performance results of this EMDB installation (also in a stress case with 8 h of vehicular signal recordings, instead of the standard case of 4).

Currently, the amount of data to be uploaded at each periodic reporting step by each experiment manager is in the order of KBs. The download, instead, may involve history data from several sites and time steps, thus in the order of hundreds of MBs. Consequently, the EMDB server, serving about 20 different users, can be safely hosted on a medium-end cloud machine.

On the other hand, more resources are demanded for the computation of the progress/performance indicators from the project’s data. But this processing can be carried out using the computational infrastructure set up at each test site for the scientific and technical goals of the project (i.e., processing the vehicular data logged to assess performance of the ADFs enhanced through the newly proposed technological enablers).

It is apparent the reduction in data size from the source data files (in the order of the GB, Table 5) to the extracted KPIs (in the order of the KB, Table 4). 

Results in Table 5 reveal that our solution efficiently handles the potentially large file sizes, allowing a rapid extraction of the needed indicators. Tests were performed on a laptop equipped with an Intel Core i7 11800 H @ 2.30 GHz processor and 16 GB of RAM.

Progress indicator extraction automation was key to reduce the reporting time and thus make the overall process acceptable for the experiment managers, who have to deal with more than 300 indicators for each reporting step. Otherwise, the number of indicators would have had to be drastically reduced, and the process would have been more error prone.

### 5.2. Instantiating the EMDB in Other Projects

The Measurify system’s code is freely available online (https://github.com/measurify/server/, accessed on 12 September 2023). The /gui sub-folder of the repository contains the code for the Admin Dashboard and of the Operation tool. All the resources are provided with instructions for installation and use.

In this sub-section, we summarize the steps necessary for third parties to instantiate their own EMDB:

1. Define the static information needed to describe each project’s experiment (i.e., the Metadata).
2. Define each experiment’s static information values (i.e., the values of the experiment Descriptor and Metadata).
3. Define the dynamic information needed to describe each project’s experiment (i.e., the Progress Indicators).
4. Define one or more Protocols to specify the data types of Metadata and Indicators.
5. Create an empty EMDB installation by using the Admin Dashboard.
6. Encode the Protocol(s) in a .json file (Figure 1), or through the Admin Dashboard GUI, without directly editing the file. Insert the protocols into the EMDB.
7. Instantiate the project’s Experiments, specifying their Descriptor, Metadata, and referenced Protocol by uploading to the server the Experiment’s .json file (Figure 2). This step can also be performed through the Admin Dashboard GUI, without directly editing the file.
8. Develop the script to automatically extract performance indicator values from the project/experiment’s data files. This step is optional, and completely project specific. It is time consuming, but highly beneficial to reduce the final reporting time.
9. Use the Operation tool for uploading and downloading the project’s history steps (Figure 4 and Figure 7, if the system includes scripts for automatic extraction of the indicators, as mentioned in the previous step).

It is important to highlight that the big data aspect of the system is project specific, so scripts need to be developed ad hoc to process the project data file (step 8 in the bullet list above). However, the EMDB may be used not only for data-intensive experiment reporting, but also for projects where the indicators for a work-package (WP) or task could be quickly manually filled on the .csv template.

### 5.3. Comparison with Off-the-Shelf Reporting Tools

Online reporting tools help users to visually analyze business information to create and share insightful business reports and dashboards. The market offers a variety of excellent tools, for instance, Zoho Analytics [32]. Power BI is a unified, scalable platform with an emphasis on business intelligence [33]. Finereport, which is free for personal use, supports the design of complex reports through simple visual drag and drop [34]. These tools (and the open source one cited below as well) expose APIs for easy integration with other services and modules (e.g., databases). All commercial tools have payable licenses, with expensive monthly/yearly service subscriptions.

In the open source world, the JasperReports Library is the most popular reporting engine. It is written in Java and able to use data from different sources and produce documents in a variety of formats [34]. The report templates for the JasperReports Library are XML files that can be edited using an Eclipse-based report designer called Jaspersoft Studio. JasperReports Server is a stand-alone and embeddable reporting server. It provides reporting and analytics that can be embedded into a web or mobile application as well as operate as a central information hub. JasperReports IO supports the related RESTful service.

While the above-mentioned open source solution is powerful and flexible, the project considered that a solution tailored to the specific needs of the project reporting would have been much more usable by the heterogeneous partnership.

The proposed solution focuses on specific requirements and offers a very simple workflow, based on the abstraction of the concepts of experiment, history steps, and protocol. It does not require any installation or editing tool (the Admin Dashboard is a simple web page with fillable fields and few buttons). This solution is much less flexible but supports a clear and common periodic reporting need, shared among projects of different types. However, extensions are possible, as the whole codebase is released online in the above-mentioned github site. On the other hand, the progress indicator extraction is project specific and should nevertheless be developed with custom code.

Finally, from a technical viewpoint, our proposed solution exploits the JavaScript-based NodeJs platform on the server side, and the high-performance MongoDB non-relational database solution for big data management, both representing leading-edge technologies.

## 6. Conclusions

As timely availability of information about the state of a project, particularly involving a large consortium with several different activities, is key for management, we explored the development of a workflow for supporting project coordinators and managers in monitoring evolution of activities in terms of pre-defined quantitatively measurable dimensions. In our work, we named project activities as experiments, but the concept is generally applicable, the key requirement being that the activity should be assessable through some quantitatively measurable dimensions that are typically provided by or extracted from a proper set of real or virtual sensors that are used for the project. The main contribution of this paper is twofold. On the one hand, it discusses how a flagship automation project exploits the big data produced by its deployed sensors to support detailed periodic reporting. On the other hand, it presents the design choices for a generic, end-to-end solution tailored to the requirements of periodic reporting. 

While our use case concerns the automotive industry, we focused our design on abstraction, with an aim being to guarantee the effectiveness of the tool in virtually any application context. By extending the Measurify framework through the Experiment and Protocol resources (and the related GUIs), we managed to make the process of setting up a MongoDB-based application just a matter of editing .json configuration files specifying the names and data format of an experiment’s progress/performance indicators.

We have identified two main user roles for the system: experiment managers, who insert all the information about their experiments, and project managers, who can read this information. While not necessary in Hi-Drive, it is possible to define finer-grained access, for instance, allowing work-package (WP) leaders to only read data about experiments of their WP.

As the quantity of data to be produced by the experiment manager at each reporting step is potentially overwhelming, and given the added value of timely reporting, some level of automation in filling in the reports is essential. To this end, it is important to make sure that most, if not all, of the quantities to be reported can be extracted from the experiment data files actually used in the project. In our use case, this allowed us to set up a workflow leading to the automatic generation of almost 90% of each history step’s report. Other fields should be added manually as they do not concern logged signals.

According to the pre-test experience by twenty partners, the new tool enables the preparation of periodic progress reports that extensively exploit actual project data.

This article focused on the development of an end-to-end periodic reporting application, the first of this kind reported in the literature, to the best of our knowledge, showing that it is possible to meet the requirements of a flagship project, in a short time, subject to the availability of a configurable tool. A straightforward next step of the research will involve a user study assessing the improvements in project management achieved through the new tool, also analyzing whether the focus on quantitatively measurable aspects really improves the quality and effectiveness of the reporting and of the project as a whole. 

We designed the EMDB abstracting functionalities to allow its use also in other projects, through simple configuration of flexible resources, such as Experiment and Protocol. Thus, other future research work will involve the testing of the developed tool in application contexts different from automated driving research projects, verifying and possibly improving the design choices.

As anticipated, the Measurify system code is freely available online (https://github.com/measurify/server/, accessed on 12 September 2023) in order to support the big data research community. 

## Figures and Tables

**Table 2:** Measurify resources.

Resource	Description
Thing	A *Thing* represents the subject (context) of a *Measurement*. A thing could be a trip, a vehicle, a house, a driver.
Feature	A *Feature* fully describes the different types of *Measurements* that can be stored in the DB. Every Measurement refers to a single Feature. Example of Features are Trip-level (or scenario instance level) performance indicators, that synthetize the performance of an ADF in an experimental vehicle’s trip (or in a segment of a trip). Each feature specifies several items, which are its constituting fields (e.g., average speed, maximum speed, travelled Kms).
Device	A *Device* is a tool providing measurements regarding a *Thing* (or an actuator that acts within a thing to modify its status).
Measurement	A *Measurement* represents a sample of a *Feature* measured by a *Device*.
Tag	A *Tag* is an alphanumerical string usable to put labels on resources, for better specifying them (e.g., to support queries, also for the dynamic generation of the graphical user interface). For instance, a measurement could be tagged with a rainy weather condition.
User	A *User* represents a user of the DB, with different roles (e.g., admin, provider, analyst).

**Table 3:** Example excerpt of a History step for the Italy1 experiment.

Field Key	Field Value
Road_dry	14
Road_wet	4
Road_icy	0
Road_snowy	2

**Table 4:** Quantitative summary of an Hi-Drive EMDB cloud installation.

Item	Value	Notes
# protocols	1	The Hi-Drive protocol only
# experiment descriptors	7	Static values set at the initialization
# metadata	88	Static values set at the initialization
# history step indicators (total)	334	
# history steps to be filled manually	41	
# experiments	20	
# history steps	18	
# hdf5 files	~40	Per experiment and history step
.hdf5 file size	~55 MB	Average, for each file
Reporting frequency	Monthly	
Cloud machine	AWS EC2 t2.medium	2 vCPU, 4 GB RAM
Upload/download speed	250–300 MBit/s	Nominal bandwidth
Upload size	~8 KB	Per experiment and history step
Download size	~500 KB	All experiments and steps
Time to upload one history step	<1 s	By an Experiment manager
Time to download all steps for one experiment	~1 s	By a Project manager
Secure protocol	https	

**Table 5:** Test results for EMDB standard and stress usage for a single experiment.

	Standard	Stress
# Data source files (.hdf5) (per experiment and step)	40	40
Signal sampling frequency	10 Hz	10 Hz
# Hours per experimental run per day	4	8
# Samples per day	144,000	288,000
# Vehicular signals sampled	21	21
Single .hdf5 file size	55 MB	110 MB
Total size of files to be processed (per experiment and step)	~2.2 GB	~4.4 GB
Progress indicator extraction time (per experiment and step)	153 s	310 s
Time to fill manual indicators	~10 min	~10 min
Time to upload one history step	<1 s	Same as standard case
Time to download all steps for one experiment	~1 s	Same as standard case

## Data Availability

Not applicable.
